# Investigation of blood flow characteristics saturated by graphene/CuO hybrid nanoparticles under quadratic radiation using VIM: study for expanding/contracting channel

**DOI:** 10.1038/s41598-023-35695-3

**Published:** 2023-05-25

**Authors:** Nidhish Kumar Mishra, Adnan, Khaleeq ur Rahman, Sayed M. Eldin, Mutasem Z. Bani-Fwaz

**Affiliations:** 1 Basic Science Department, College of Science and Theoretical Studies, Saudi Electronic University, Riyadh, 11673 Saudi Arabia; 2 Department of Mathematics, Mohi-ud-Din Islamic University, Nerian Sharif, 12080 AJ&K Pakistan; 3 Faculty of Engineering, Center of Research, Future University in Egypt, New Cairo, 11835 Egypt; 4 Department of Chemistry, College of Science, King Khalid University, P. O. Box 9004, Abha, 61413 Saudi Arabia

**Keywords:** Engineering, Mathematics and computing, Nanoscience and technology

## Abstract

The importance of heat transfer in nanoliquids cannot avoided because it playing crucial role in the applied research fields. The potential area of applications included but restricted to applied thermal, biomedical, mechanical and chemical engineering. Therefore, it is the need of time to introduce new efficient way to enhance the heat transport rate in common fluids. The major aim of this research is to develop a new heat transport BHNF (Biohybrid Nanofluid Model) model in a channel having expanding/contracting walls up to Newtonian regimes of blood. The two sort of nanomaterials (Graphene + CuO) along with blood as base solvent are taken for the formation of working fluid. After that, the model analyzed via VIM (Variational Iteration Method) to examine the influence of involved physical parameters on the behavior of bionanofluids. The model results revealed that the bionanofluids velocity rises towards the lower and upper channel end when the expanding/contracting of the walls in the range of 0.1–1.6 (expanding case) and $- \, 0.1$ to $1.6$ (contraction case). The working fluid attained high velocity in the neighboring of center portion of the channel. By increasing the walls permeability (${A}_{1}=\text{0.1,0.2,0.3,0.4}$), the fluid movement can be reduced and optimum decrement observed about $\eta =0.0$. Further, inclusion of thermal radiation (R_d_) and temperature coefficient (${\theta }_{r}$) observed good to enhance thermal mechanism in both hybrid and simple bionanofluids. The present ranges of R_d_ and ${\theta }_{r}$ considered from $0.1$ to $0.9$ and $0.1$ to $1.4$, respectively. Thermal boundary layer reduced in the case of simple bionanoliquid keeping ${P}_{r}=21.0$.

## Introduction

The analysis of blood dynamics in human body is one of the interesting research area. Blood has different functions inside the body which include transportation of oxygen to the lungs, formation of blood clots to prevent the excessive blood loss, maintain body temperature, formation of antibodies that fight against infection and transport of waste products to the liver and kidney for the blood filtration. The human circulatory system is very important that facilitate the transportation of different essential components like metabolic toxins, oxygen and hormones etc. to the other body parts. This system contains three key components namely blood vessels, heart and blood. The uniform expansion and contraction of heart and vessels supply the blood to the other parts for various functions.

The role of nanoparticles in the study of blood dynamics is essential. Induction of various nanoparticles (Graphene, CuO, Al_2_O_3_ etc.) in blood use to diagnose various diseases in human body. The different nanoparticles use for different purposes like cancer detection and cancer therapy etc. The nanoparticles consistently injected in the vessels that target the desired cells inside the body which then detected by magnetic resonance imaging (MRI) after receiving specific band signal. Further, Graphene nanoparticles are helpful to targeted drug delivery, gene delivery, tissue engineering and in phototherapy etc.

Hybrid nanofluid is an extended version of conventional nanofluids. These fluids prepare using two different sort of nanoparticles in various basic fluids like water, ethylene glycol, blood and kerosene oil etc. Due to addition of two distinct nanoparticles (see Refs.^1–3^), thermal conductivity of resultant hybrid fluid improves significantly which ultimately contributes in the heat transport mechanism. These superior characteristics make such fluids more effective and thus strengthen their roots in engineering and industrial applications.

The researchers and engineers made efforts to analyze the performance of hybrid nanofluids (See Refs.^4–6^) with addition of physical constraints. In 2017, Singh et al.^7^ studied mass and heat transfer between two parallel plates for squeezing unsteady nanofluid flow by considering the effects of slip velocity and uniform magnetic field and inspected that the mass transport at the walls increased for increasing squeezed number. Saeed et al.^8^ examined the heat transfer and irreversible behavior for couple stress hybrid nanofluid^9^ over an extended surface. The hybrid nanofluid^10^ is composed of human blood in pure form with nanoparticles of MWCNTs and SWCNTs. They used HAM (Homotopy analysis method) for mathematical investigation of the model and reported that the temperature rises with increase in radiation factors. The study of hybrid nanofluids in channel form by two permeable walls gained huge attention of the researchers and engineers. Therefore, Alghamdi et al.^11^ discussed the study about hybrid nanofluid^12,13^ in a rectangular domain between two permeable channels and considered blood as primary fluid. They preferred HAM (Homotopy Analysis Method) approach for the solution purpose and noticed considerable variations in the momentum and temperature of the considered fluid. Hosseinzadeh and Ganji^14^ analyzed the mass and heat transmission mechanism in steady magnetohydrodynamic nanofluid with insertion of hybrid nanoparticles^15^. The working domain preferred between two parallel plates under consistent magnetic field. The model analyzed numerically and concluded that the Nusselt number decreased with increase in thermophoretic and Brownian parameters. Some recent trends on the analysis of nano and bihybrid nanofluids reported by various researchers (see Refs.^16–18^) and found these fluids as useful tool for thermal enhancement applications.

The non-Newtonian nanofluids are also of much interest due to their heating/cooling performance and widespread uses in multiple engineering and industrial areas. Recently, the researchers (see Refs.^19–22^) paid efforts to introduce new nanofluid models of non-Newtonian nature based on their stress tensor. Recently, Hussain and Khan^23^ reported the influence of thermal stratification with additional effects of activation energy in the heat transport performance of polymer based nanoliquid. They found that Biot and Schmidt numbers are good physical effects to enhance and reduce the heat and mass transport in nanoliquids. Another significant investigation of Bioconvection Casson nanoliquid have been done by Waqas et al.^24^. The authors focused on the effects of diffusion gradients and analyzed the model via analytical scheme.

Xiu et al.^25^ reported the dynamics of ternary nanoliquids over an elastic wedge surface. They analyzed the fluid characteristics by keeping low and high concentration factor and also included the phenomena of forced convection. From the physical results, they deduced that the shear drag rate improved up to 0.07 and reduced to 0.008 for small and large concentration ranges, respectively. Similarly, Animasaun et al.^26^ and Cao et al.^27^ discussed the nontransient characteristics of water based nanoliquids by considering the small and larger weight concentration of the nanoparticles, effects of stretching and partial slip. They concluded that addition of ternary nanoparticles in the Newtonian way is a reliable method to enhance the thermal transmission which is important from industrial applications. The most related studies in the direction of nanoliquids under the variety of controlling parameters for various flow configuration discussed by different researchers (see Refs.^28–33^).

In 2016, Sheikholeslami et al.^34^ studied the behavior of steady nanofluid flow. The analytical scheme DTM (Differential Transform Method) used to solve the model. They found that the skin fraction increased with augmentation in viscosity and magnetic parameters increases. Yaseen et al.^35^ presented the model in which upper plate is moving towards the lower plate with linear velocity. The model solved by using the bvp4c function and investigated the control of various factors such as temperature, heat transfer rate, streamlines and velocity boundary layer. In 2017, Hosseinzadeh et al.^36^ investigated an MHD two phase nanofluid model. The authors preferred three distinct methods (Finite Element, Collocation, Homotopy Perturbation) for the model analysis. The outcome shown that the temperature profile increased with increasing Brownian parameter. Famakinwa et al.^37^ have done the analysis of thermal transport under thermal radiation and viscous dissipation on unsteady incompressible squeezing flow between two parallel plates. They considered (CuO–Al_2_O_3_)/w^38,39^ as a working fluid with variable viscosity. Khashiie et al.^40^ studied Cu–Al_2_O_3_/water model and used numerical scheme to inspect the model dynamics. They examined that injection, squeeze and magnetic parameters result to the decrement in the temperature at the bottom plate.

In 2020, Salehi et al.^41^ studied a single phase heat transfer model using hybrid nanoparticles. The AGM (Akbari-Ganji method) used to solve the model and furnished the physical results. They analyzed the effects of squeeze number and Harman number. The results of the study showed that the rise in Harman number and Squeeze number causes the decline in velocity profile. Rauf et al.^42^ investigated the micropolar tri-hybrid nanoliquid model for heat transfer applications under MHD effects. Sheikholeslami et al.^43^ investigated the nanofluid flow of unsteady squeezing flow between two parallel plates by using HPM.

As, in the open literature, many numerical as well as analytical schemes (see Refs.^44,45^) are available which have been used to investigate the heat transport problems including the Newtonian, non-Newtonian and Nanofluids. Each scheme has their own advantages or disadvantages in the view of model nonlinearity. However, the Variational Iteration Method^46^ is one of the powerful mathematical tool which convinced the researchers in last few decades. Primarily, this scheme works for both weakly strongly linear and nonlinear nanofluid models. The main reason behind the selection of this scheme is their less computational cost and meaningful accuracy. The VIM based on the Lagrange multiplier and initial guesses of the model which are easy to compute for more accurate results of the model.

From the cited literature, it is investigated that the previous efforts have been done for simple mono nanofluids by considering blood as base solvent in the absence of nonlinear radiation and absorptivity effects which need to be addressed. Therefore, the present analysis carried out to cover this significant gap. The study will prolong to hybrid nanoliquid which is extended version of the simple mono nanoliquid.

The salient features of the present analysis will to examine the behaviour of blood dispersed with hybrid nanoparticles influenced by significant physical constraints. The model will develop via effective characteristics of nanoparticles and blood and to prolong it for more fascinating results, the important effects of radiation factor and the walls permeability introduced. Thus, the working model will be achieved in the form of ODEs (Ordinary Differential Equations) which then handled through analytical scheme called VIM (Variational Iteration Method). As, many other schemes (like HAM, RK, GFEM and VPM) are available in the literature to analyze the nonlinear models. However, the VIM is suitable for the solution of the present problem. After the successful implementation of the VIM, the model results furnished for the various parametric values and will discuss in-depth.

This work will covers the answer to the following research questions which are important to discuss:

- How the velocity of blood base hybrid nanoliquid can be controlled in a channel under absorptivity property?
- How the quadratic radiations impact the heat transport mechanism in bionanofluid due to expanding and contraction of the channel boundaries?
- In which region the fluid will attain optimum or minimal velocity and temperature under the physical constraints?
- Is the VIM is suitable to analyze the nonlinear nanoliquid models with finite boundaries?

## Model development

### Model statement and geometry

**Figure 1:**
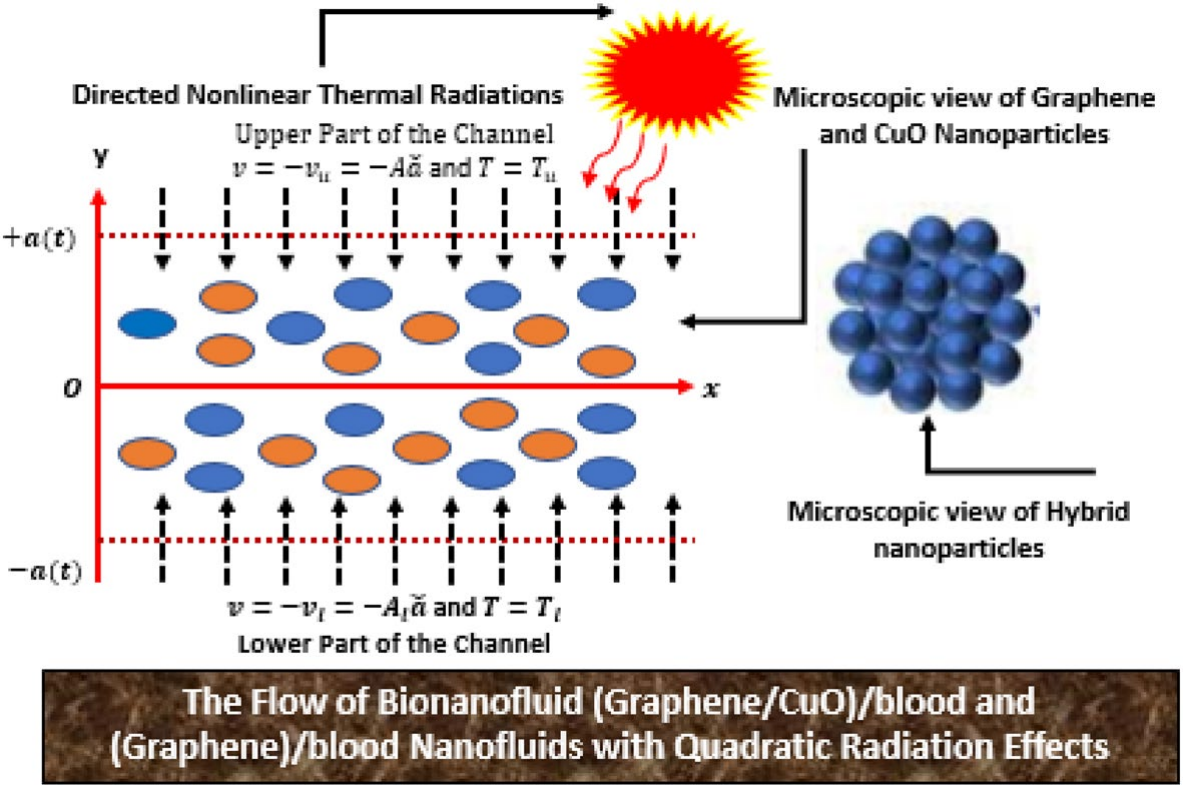
The bionanofluids (Graphene–CuO)/blood and (Graphene)/blood nanofluids.

The hybrid nanofluid flow between two parallel permeable plates is considered and the upper plate is fixed at $y=a(t)$ and lower plate at $y=- \, a(t)$.The components of velocity are *u* and *v* designated in the direction of x and y, respectively. To achieve the desired hybrid nanofluid model, the governing equations updated including the effects of hybrid nanofluid, and thermal radiations. It is assumed that the flow is in laminar flow regimes, incompressible, viscous and no chemical reaction take place inside the fluid. Further, the Graphene and CuO nanoparticles dispersed in blood uniformly. Moreover, ${T}_{l}$ and ${T}_{u}$ designated as temperature at the channel ends and $-{ \, v}_{u}$ and ${- \, v}_{l}$ are the consistent permeability of the walls. Addition of nonlinear thermal radiation aspect in the governing model lead to the appearance of radiation and temperature ratio number which are excellent to enhance the temperature of the model. The appropriate similarity transforms will be adopted for the model development. The physical configuration for biohybrid nanoliquid is shown in Fig. 1.

Keeping in view the above flow assumptions, the governing equations are constructed as:

1 $$\frac{\partial \widetilde{u}}{\partial x}+\frac{\partial \widetilde{v}}{\partial y}=0,$$

2 $${\rho }_{biohnf}\left( \frac{\partial \widetilde{u}}{\partial t}+\widetilde{u}\frac{\partial \widetilde{u}}{\partial \widetilde{x}}+\widetilde{v}\frac{\partial \widetilde{u}}{\partial \widetilde{y}} \right)=-\frac{\partial \widetilde{P}}{\partial \widetilde{x}}+{\mu }_{biohnf}\left[ \frac{{\partial }^{2}\widetilde{u}}{\partial {\widetilde{x}}^{2}} +\frac{{\partial }^{2}\widetilde{u}}{\partial {\widetilde{y}}^{2}} \right],$$

3 $${\rho }_{biohnf}\left( \frac{\partial \widetilde{v}}{\partial t}+\widetilde{u}\frac{\partial \widetilde{v}}{\partial \widetilde{x}}+\widetilde{v}\frac{\partial \widetilde{v}}{\partial \widetilde{y}} \right)=-\frac{\partial \widetilde{P}}{\partial \widetilde{y}}+{\mu }_{biohnf}\left[ \frac{{\partial }^{2}\widetilde{v}}{\partial {\widetilde{x}}^{2}} +\frac{{\partial }^{2}\widetilde{v}}{\partial {\widetilde{y}}^{2}} \right],$$

4 $${\left(\rho {C}_{p}\right)}_{biohnf}\left( \frac{\partial \widetilde{T}}{\partial t}+\widetilde{u}\frac{\partial \widetilde{T}}{\partial \widetilde{x}}+\widetilde{v}\frac{\partial \widetilde{T}}{\partial \widetilde{y}} \right)={k}_{biohnf}\left[ \frac{{\partial }^{2}\widetilde{T}}{\partial {\widetilde{x}}^{2}} +\frac{{\partial }^{2}\widetilde{T}}{\partial {\widetilde{y}}^{2}} \right]+\left[\frac{\partial }{\partial \widetilde{x}}+\frac{\partial }{\partial \widetilde{y}}\right]\frac{4{\sigma }^{*}{\widetilde{T}}^{3}}{3{\widetilde{a}}_{k}} \frac{\partial \widetilde{T}}{\partial \widetilde{y}}.$$

The accompanying BCs for the bionanofluids model in dimensional versions are:

5 $$\left\{ \begin{array}{c}\widetilde{u}=0, T={T}_{l }, \widetilde{v}=-{v}_{l}=-h{A}_{l}\, at\, \widetilde{y} = - h(t)\\ \widetilde{u}=0, T={T}_{u} ,\widetilde{v}{=-v}_{u}=-h{A}_{u} \,at\, \widetilde{y}= h(t)\end{array}\right..$$

Now, here we introduce;

6 $$\widetilde{\chi }=\frac{\partial \widetilde{v}}{\partial \widetilde{x}}-\frac{\partial \widetilde{u}}{\partial \widetilde{y}}.$$

To eliminate $P(x,y)$ from Eqs. (2) and (3), it is essential to perform cross differentiation and then necessary mathematical action yields the below version of the model equation.

7 $${\rho }_{biohnf}\left(\frac{\partial \widetilde{\chi }}{\partial t}+\widetilde{u}\frac{\partial \widetilde{\chi }}{\partial \widetilde{x}}+\widetilde{v}\frac{\partial \widetilde{\chi }}{\partial \widetilde{y}}\right)={\mu }_{biohnf}\left[\frac{{\partial }^{2}\widetilde{\chi }}{\partial {\widetilde{x}}^{2}}+\frac{{\partial }^{2}\widetilde{\chi }}{\partial {\widetilde{y}}^{2}}\right].$$

The outcomes are:

8 $${\rho }_{biohnf}\left(\frac{{\partial }^{2}\widetilde{u}}{\partial t\partial y}+\widetilde{u} \frac{{\partial }^{2}\widetilde{u}}{\partial x\partial y} +\widetilde{v} \frac{{\partial }^{2}\widetilde{u}}{\partial {y}^{2}}\right)={\mu }_{biohnf }\frac{{\partial }^{3}\widetilde{u}}{\partial {y}^{3}}.$$

Now, at his stage introducing the subsequent transformations parameters further reduced our model expression into simpler form:

9 $$y=\eta h,$$

10 $$\widetilde{u}=\frac{{\upsilon }_{f \widetilde{x} {\widetilde{f}}_{\eta }}}{{h}^{2}}, \widetilde{v}=-\frac{{\upsilon }_{f \widetilde{f }\left(\eta ,t\right)}}{h} , \widetilde{\psi }=-\frac{{\upsilon }_{f \widetilde{x} \widetilde{f }\left(\eta ,t\right)}}{h} , \theta \left(\eta \right)=\frac{\widetilde{T }- {\widetilde{T}}_{u} }{{\widetilde{T}}_{i}-{\widetilde{T}}_{u}}.$$

Using the transformative parameters from Eq. (10), the BCs reduced in the below version:

11 $$\left\{\begin{array}{c}\frac{\partial \widetilde{f }}{\partial \eta }=0,\widetilde{f }={Re}_{ , }Re=\frac{{h{h}^{.}A}_{u}}{{\upsilon }_{f}}\, at\, \eta =-1\\ \frac{\partial \widetilde{f }}{\partial \eta }=0,\widetilde{f}={Re}_{ , }Re=\frac{{h{h}^{.}A}_{u}}{{\upsilon }_{f}}\, at\, \eta =1\end{array}\right..$$

It is worth stating that the Reynolds number (Re) is associated with suction/injection due to its positive or negative values. Now, we need to introduce a second set of transformative quantities to make the model easier.

12 $$u=\frac{\widetilde{u}}{h},v=\frac{\widetilde{v}}{h},x=\frac{\widetilde{x}}{h},f=\frac{\widetilde{f}}{Re}.$$

### Thermo-physical attributes for hybrid nanofluids

Inspired by the thermophysical attributes of spherical and cylindrical nanoparticles, the following correlations used to obtain the resultant (Graphene–CuO)/blood and (Graphene)/blood nanofluids. It is obvious that the below expressions derived for spherical and cylindrical nanoparticles.

**Density of bionanofluids**:

$${\rho }_{biohnf}=\left(1-{\phi }_{G}-{\phi }_{\text{CuO}}\right){\rho }_{blood}+{\phi }_{G}{\rho }_{G}+{\phi }_{2}{\rho }_{\text{CuO}}.$$

**Heat capacity of bionanofluids**:

$${\left(\rho {C}_{p}\right)}_{biohnf}=\left(1-{\phi }_{G}-{\phi }_{\text{CuO}}\right){\left(\rho {C}_{p}\right)}_{blood}+{\phi }_{G}{\left(\rho {C}_{p}\right)}_{G}+{\phi }_{\text{CuO}}{\left(\rho {C}_{p}\right)}_{\text{CuO}}.$$

**Dynamic viscosity of bionanofluids**:

The viscosity models of nanofluid having different particle shapes are described below in which SP and CP indicate the Spherical and Cylindrical nanoparticles, respectively.

$${\left({\mu }_{bloodnf}\right)}_{G}={\mu }_{blood}\left(1+2.5\phi +6.2{\phi }^{2}\right) \quad \text{For SP},$$

$${\left({\mu }_{bloodnf}\right)}_{\text{CuO}}={\mu }_{blood}\left(1+13.5\phi +904.5{\phi }^{2}\right) \quad \text{ For CP},$$

$${\mu }_{biohnf}=\frac{{{\left({\mu }_{bionf}\right)}_{G}\phi }_{G}+{{\left({\mu }_{bionf}\right)}_{\text{CuO}}\phi }_{\text{CuO}}}{\phi }.$$

**Thermal conductivity of bionanofluids**:

Thermal conductivity of BHNF is computed by using ${\left({k}_{bloodnf}\right)}_{G}$ and ${\left({k}_{bionf}\right)}_{\text{CuO}}$. The resultant BHNF expression is given in the subsequent equations. Further, $\phi$ is the average of Graphene and CuO nanoparticles.

$${\left({k}_{bloodnf}\right)}_{G}=\frac{{k}_{G}+2{k}_{blood}+2{\phi }_{G}({k}_{G}-{k}_{blood})}{{k}_{G}+2{k}_{blood}-{\phi }_{G}({k}_{G}-{k}_{blood})}\times {k}_{blood}\quad \text{For SP }\left(\text{spherical particles}\right),$$

$${\left({k}_{bionf}\right)}_{\text{CuO}}=\frac{{k}_{\text{CuO}}+3.9{k}_{blood}+3.9{\phi }_{\text{CuO}}({k}_{\text{CuO}}-{k}_{blood})}{{k}_{\text{CuO}}+3.9{k}_{blood}-{\phi }_{\text{CuO}}({k}_{\text{CuO}}-{k}_{blood})}\times {k}_{blood} \quad \text{For CP }\left(\text{cylindrical particles}\right),$$

$$\frac{{k}_{biohnf}}{{k}_{blood}}=\frac{{\left({k}_{bionf}\right)}_{G}+{\left({k}_{bionf}\right)}_{\text{CuO}}}{\phi }\quad \text{For Biohybrid nanofluids }\left(\text{BHNF}\right).$$

It is significant to add the values of these bionanofluids characteristics because these greatly contribute in the dynamics of bionanofluids. For this, Table 1 organized which indicates the particular values of these factors. Further, the value of Prandtl number $\left({P}_{r}=\frac{{\nu }_{f}{\left(\rho {c}_{p}\right)}_{f}}{{k}_{f}}\right)$ is taken as $21.0$.

**Table 1:** The particular values of Thermophysical factors for the formation of biohybrid nanofluid model^47^.

Working fluid/nanoparticles	Characteristics			
	$\rho$	${C}_{p}$	$k$	$\mu$
Blood	$1063$	$3594$	$0.492$	$2.9\times {10}^{-3}$
Graphene	$2200$	$790$	$5000$	–
CuO	$6500$	$535.6$	$20$	–

### The biohybrid nanofluid model

After performing the complete mathematical procedure using transformative factors and the thermophysical attributes of the functional fluid blood and SP, CP nanoparticles, the following version obtained:

13 $${F}{{^{\prime\prime\prime\prime}}} +\frac{{\rho }_{\frac{biohnf}{{\rho }_{blood}}}}{{\mu }_{\frac{biohnf}{{\mu }_{blood}}}}\left(\left(\eta {F}^{{^{\prime\prime\prime\prime}}}+3{F}^{{{\prime\prime}}}\right){\alpha }_{1}-{R}_{1}\left({F}^{{\prime}}{F}^{\prime\prime}-F{F}^{\prime\prime\prime}\right)\right)=0,$$

14 $$F\left(\eta l\right)={A}_{1},{F}^{^{\prime}}\left(\eta l\right)=0,F\left(\eta u\right)=1,{F}^{^{\prime}}\left(\eta u\right)=0.$$

In Eq. (14), $\eta l=0.0$ and $\eta u=1.0$ designates the bottom and top wall conditions. The converted energy equation is obtained by using transformations:

15 $$\frac{{K}_{biohnf}}{{k}_{blood}}\beta^{\prime\prime}+\frac{{\text{P}}_{\text{r}}{\left(\uprho {\text{C}}_{\text{p}}\right)}_{\text{biohnf}}}{{\left(\rho {c}_{p}\right)}_{blood}}\left({\alpha }_{1}\eta +{R}_{1}F\right){\theta }^{^{\prime}}-{R}_{d}({\left(1+\left({\theta }_{r}-1\right)\beta \right)}^{3}{\beta }^{{{\prime\prime}}}+3{\left(1+\left({\theta }_{r}-1\right)\beta \right)}^{2}\left({\beta {^{\prime}})}^{2}\left({\theta }_{r}-1\right)\right)=0,$$

16 $$\beta \left(-1\right)=1, \beta \left(1\right)=0.$$

### Skin friction and nusselt number

The drag force study and local thermal gradient is very impressive topic in the study of bionanofluids. For the current model, these expressions defined in the subsequent equations:

17 $${C}_{F}=\frac{\tau a\left(t\right)}{{\rho }_{biohnf}{v}_{l}^{2}}, {\tau }_{w}={\mu }_{biohnf}\left[\frac{\partial \widetilde{u}}{\partial y}\right]\text{ when }y=\mp a,$$

18 $${N}_{u}=\frac{a}{{k}_{f}({T}_{l}-{T}_{u})}\left[\frac{{k}_{biohnf}\partial T}{\partial y}+{q}_{rd}\right]\text{ when }y=\mp a.$$

Using the biohybrid nanofluid expressions along with the transformative factors, the following dimensionless formulae achieved:

19 $${R}_{l}^{2}{C}_{Fl}=\frac{\left[\frac{{{\left({\mu }_{bionf}\right)}_{G}\phi }_{G}+{{\left({\mu }_{bionf}\right)}_{\text{CuO}}\phi }_{\text{CuO}}}{\phi }\right]}{\left[\left(1-{\phi }_{G}-{\phi }_{\text{CuO}}\right)+\frac{{\phi }_{G}{\rho }_{G}}{{\rho }_{blood}}+\frac{{\phi }_{2}{\rho }_{\text{CuO}}}{{\rho }_{blood}}\right]}{F}^{{{\prime\prime}}}\left(-1\right),$$

20 $${R}_{u}^{2}{C}_{Fu}=\frac{\left[\frac{{{\left({\mu }_{bionf}\right)}_{G}\phi }_{G}+{{\left({\mu }_{bionf}\right)}_{\text{CuO}}\phi }_{\text{CuO}}}{\phi }\right]}{\left[\left(1-{\phi }_{G}-{\phi }_{\text{CuO}}\right)+\frac{{\phi }_{G}{\rho }_{G}}{{\rho }_{blood}}+\frac{{\phi }_{2}{\rho }_{\text{CuO}}}{{\rho }_{blood}}\right]}{F}^{{{\prime\prime}}}\left(1\right),$$

21 $${N}_{ul}=\left|{\beta }^{{\prime}}\left(-1\right)\left(\frac{{\left({k}_{bionf}\right)}_{G}+{\left({k}_{bionf}\right)}_{\text{CuO}}}{\phi }\right)+{R}_{d}{\left(1-\left(1-{\theta }_{r}\right)\beta \left(-1\right)\right)}^{3}\right|,$$

22 $${N}_{up}=\left|{\beta }^{{\prime}}\left(1\right)\left(\frac{{\left({k}_{bionf}\right)}_{G}+{\left({k}_{bionf}\right)}_{\text{CuO}}}{\phi }\right)+{R}_{d}{\left(1-\left(1-{\theta }_{r}\right)\beta \left(1\right)\right)}^{3}\right|.$$

## Mathematical analysis

The under consideration bionanofluid model is comprises nonlinearities due to the directed physical situations. For such models, different computational schemes like neural network, HAM, RK^48,49^, GFEM (Galerkin Finite Element Method)^50–53^ and VPM (Variation of Parameters) etc. have been exercised by different researchers (see Refs.^54–56^). It is evident that each scheme has their own limitations and advantages according to the nature of working model. Thus, for the present model, an analytical scheme based on Lagrange multiplier is selected because of their suitability. This technique is easy to implement with less computational cost. Therefore, the model is solved via VIM Variational Iteration Method (VIM) to examine the real behaviour of the dynamics of the (Graphene–Ag)/blood. This technique is reliable to analyze such models. The VIM works according to the following major steps.

### Arrangement of the model

Before going to start the technique, it is essential to set the model in the following standard format.

23 $${\overbrace{\mathcal{L}}}_{1}F+{\overbrace{\mathfrak{R}}}_{1}F+{\overbrace{\aleph }}_{1}F+{\overbrace{q}}_{1}^{*}\left(\eta \right)=0,$$

24 $${\overbrace{\mathcal{L}}}_{2}\beta +{\overbrace{\mathfrak{R}}}_{2}\beta +{\overbrace{\aleph }}_{2}\beta +{\overbrace{q}}_{2}^{*}=0.$$

It is understood that the function $F$ and $\beta$ purely depend on the $\eta$. Further, the terms comprising in Eqs. (23) and (24) are highest order linear operators (${\overbrace{\mathcal{L}}}_{1}, {\overbrace{\mathcal{L}}}_{2}$), linear operators (${\overbrace{\mathfrak{R}}}_{1}, {\overbrace{\mathfrak{R}}}_{2}$), nonlinearities (${\overbrace{\aleph }}_{1}, {\overbrace{\aleph }}_{2}$) and nonhomogeneous parts (${\overbrace{q}}_{1}^{*},{\overbrace{q}}_{2}^{*}$).

### Defining lagrange multiplier

After the model arrangement in the standard way, now we define suitable Lagrange multiplier for the model according to its order. These multiplier for the current model situation are as follows:

25 $${\uplambda }_{F}=\frac{{\left(-1\right)}^{n*}{\left(\upeta -\text{s}\right)}^{{n}^{*}-1}}{\left({n}^{*}-1\right)!},$$

26 $${\uplambda }_{\beta }=\frac{{\left(-1\right)}^{n*}{\left(\upeta -\text{s}\right)}^{{n}^{*}-1}}{\left({n}^{*}-1\right)!}.$$

Here, ${n}^{*}$ indicates the order of the model and ${\uplambda }_{F}$ and ${\uplambda }_{\beta }$ are the associated multipliers which help for the solution computation. According to the model order, the Lagrange multiplier enlisted in Eqs. (25) and (26) take the subsequent form.

27 $${\uplambda }_{F}=\frac{{\left(-1\right)}^{4}{\left(\upeta -\text{s}\right)}^{3}}{3!},$$

28 $${\uplambda }_{\beta }=\frac{{\left(-1\right)}^{2}{\left(\upeta -\text{s}\right)}^{1}}{1!}.$$

### Selection of initial guess

The correct form of the initial guesses can be obtained by keeping in mind the model order and initial conditions defined for it. Thus, it take the following form in which “$i$” varies from $0$ to the model order “*j*”.

$${F}_{0}=\sum_{i=0}^{j}\frac{{\eta }^{i}}{i!}\frac{{d}^{i}}{d\eta }\left[F\left(0\right)\right]\text{ and }{\beta }_{0}=\sum_{i=0}^{j}\frac{{\eta }^{i}}{i!} \frac{{d}^{i}}{d\eta } \left[\beta (0)\right].$$

By expanding the above sigma notation up to the order of the model equations, below expressions obtained:

$${F}_{0}=F\left(0\right)+\frac{\eta {F}^{{\prime}}\left(0\right)}{1!}+{\eta }^{2}\frac{{F}^{{{\prime\prime}}}\left(0\right)}{2!}+{\eta }^{3}\frac{{F}^{{{\prime\prime\prime}}}\left(0\right)}{3!},$$

$${\beta }_{0}=\beta \left(0\right)+\frac{\eta {\beta }^{{\prime}}\left(0\right)}{1!}.$$

By exercising the initial conditions in ${F}_{0}$ and ${\beta }_{0}$, we will acquire the desired IG (Initial Guess) of the model:

$${F}_{0}={A}_{1}+{\eta }^{2}\frac{{\Gamma }_{1}}{2!}+{\eta }^{3}\frac{{\Gamma }_{2}}{3!} \text{ and }{\beta }_{0}={\Gamma }_{3}+\frac{\eta {\Gamma }_{4}}{1!}.$$

Here, ${\Gamma }_{i}$ for $i=\text{1,2},\text{3,4}$ are the unknown against ${F}^{{{\prime\prime}}}\left(0\right), F{^{\prime\prime\prime}}(0), \beta (0)$ and ${\beta }^{^{\prime}}\left(0\right)$, respectively. For computation these, the remaining boundary conditions used.

### Final solution of the model

After the operations performed in Step 1 to Step 3, the following solution of the obtained for the biohybrid nanofluid model.

$${F}_{n+1}={F}_{0}+\underset{{\eta }_{l}}{\overset{{\eta }_{u}}{\int }}{\lambda }_{F}(-{\overbrace{\mathfrak{R}}}_{1}F\left(s\right)-{\overbrace{\aleph }}_{1}F\left(s\right)-{\overbrace{q}}_{1}^{*}\left(s\right))ds, n\ge 0,$$

$${\beta }_{n+1}={\beta }_{0}+\underset{{\eta }_{l}}{\overset{{\eta }_{u}}{\int }}{\lambda }_{\beta }(-{\overbrace{\mathfrak{R}}}_{2}\beta \left(s\right)-{\overbrace{\aleph }}_{2}\beta \left(s\right)-{\overbrace{q}}_{2}^{*}\left(s\right))ds, n\ge 0.$$

Adding the initial guesses (${F}_{0}$ and ${\beta }_{0}$) and the Langrage multiplier in above recursive relation for the velocity and temperature, the following modified form obtained:

$${F}_{n+1}={A}_{1}+{\eta }^{2}\frac{{\Gamma }_{1}}{2!}+{\eta }^{3}\frac{{\Gamma }_{2}}{3!} +\underset{{\eta }_{l}}{\overset{{\eta }_{u}}{\int }}\frac{{\left(-1\right)}^{4}{\left(\upeta -\text{s}\right)}^{3}}{3!}(-{\overbrace{\mathfrak{R}}}_{1}F\left(s\right)-{\overbrace{\aleph }}_{1}F\left(s\right)-{\overbrace{q}}_{1}^{*}\left(s\right))ds, n\ge 0,$$

$${\beta }_{n+1}={\Gamma }_{3}+\frac{\eta {\Gamma }_{4}}{1!}+\underset{{\eta }_{l}}{\overset{{\eta }_{u}}{\int }}\frac{{\left(-1\right)}^{2}{\left(\upeta -\text{s}\right)}^{1}}{1!}(-{\overbrace{\mathfrak{R}}}_{2}\beta \left(s\right)-{\overbrace{\aleph }}_{2}\beta \left(s\right)-{\overbrace{q}}_{2}^{*}\left(s\right))ds, n\ge 0.$$

Now, the successive iteration of the scheme computed by increasing the index $n$ in the above formulas, which complete the solution procedure.

## Results and discussion

### The velocity of biohybrid nanofluid with changing parameters

**Figure 2:**
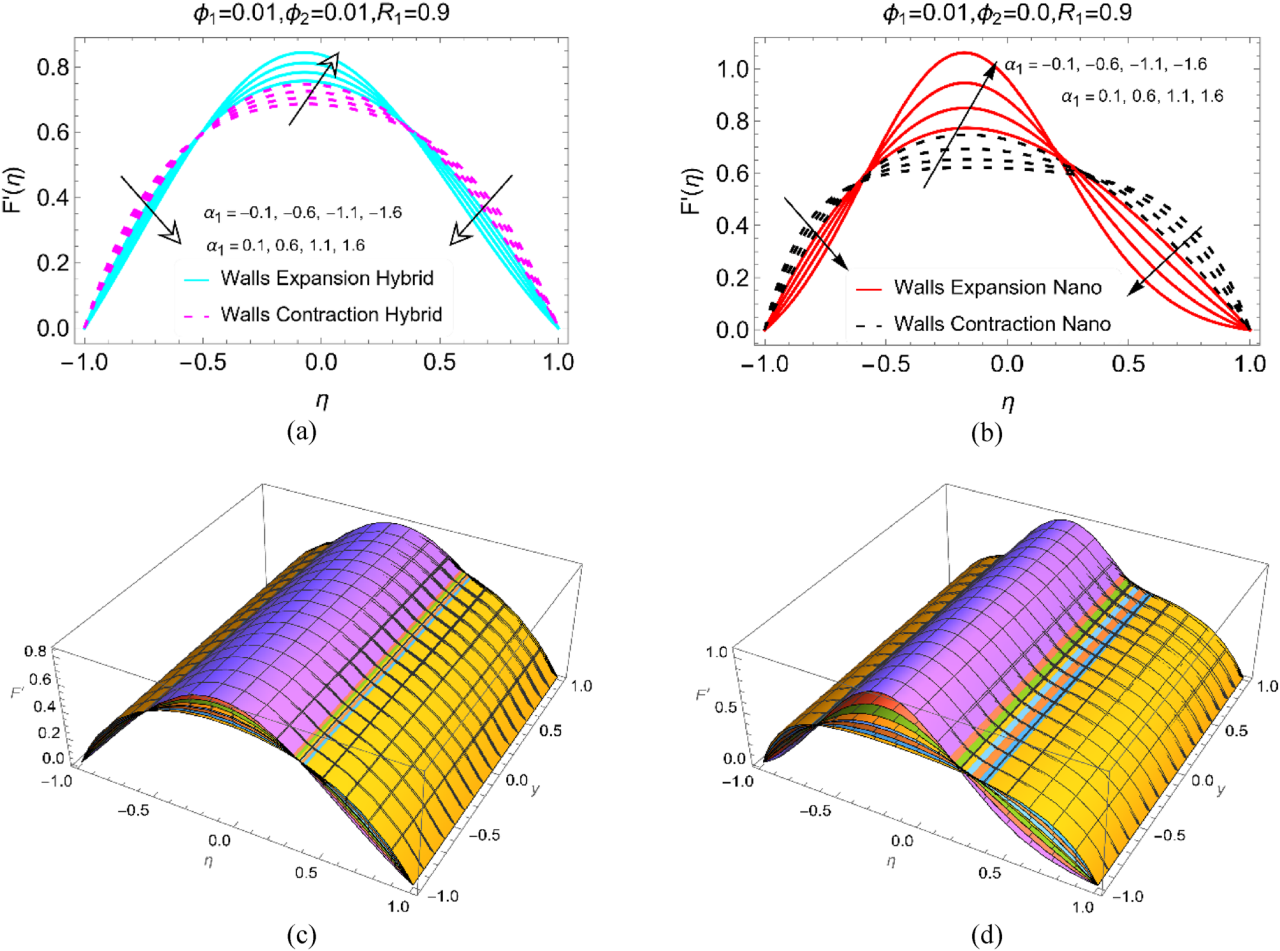
The velocity $\text{F}{^{\prime}}(\upeta )$ against ${{\upalpha }}_{1}$ for (**a**) Hybrid, (**b**) Nano and 3D view for (**c**) Hybrid and (**d**) Nano.

Figure 2a,b emphasis on the analysis of velocity distribution for BHNF and BHF while the parameter ${\alpha }_{1}$ varies in the range of $- \, 0.1$ to $- \, 1.6$ (contraction case) and $0.1$ to $1.6$ (expanding case). Figure 2a demonstrating that the velocity of BHNF vanishes towards the channel walls by keeping the permeable number $0.3$. The velocity of BHNF is optimum near $\eta = 0.0$ and it is zero near the channel walls. For expansion case, the BHNF moves rapidly than contracting case. Physically, expansion of the walls provided maximum working domain in which the BHNF particles flow freely. The adverse pressure in the neighboring of walls act in backward direction and opposes the motion. While focusing on Fig. 5b, it can be seen that the simple BNF movement is rapid than that of BHNF for both expansion/contraction cases. Physically, the BNF is less dense than that of BHNF, the fluid particles move quickly because of weaker viscous forces in BNF. Further, the quite prominent movement near the walls is observed for BNF case. Figure 2c,d highlighting the 3D scenarios of Fig. 2a,b. The value of Prandtl number is used as ${P}_{r}=21.0$.

**Figure 3:**
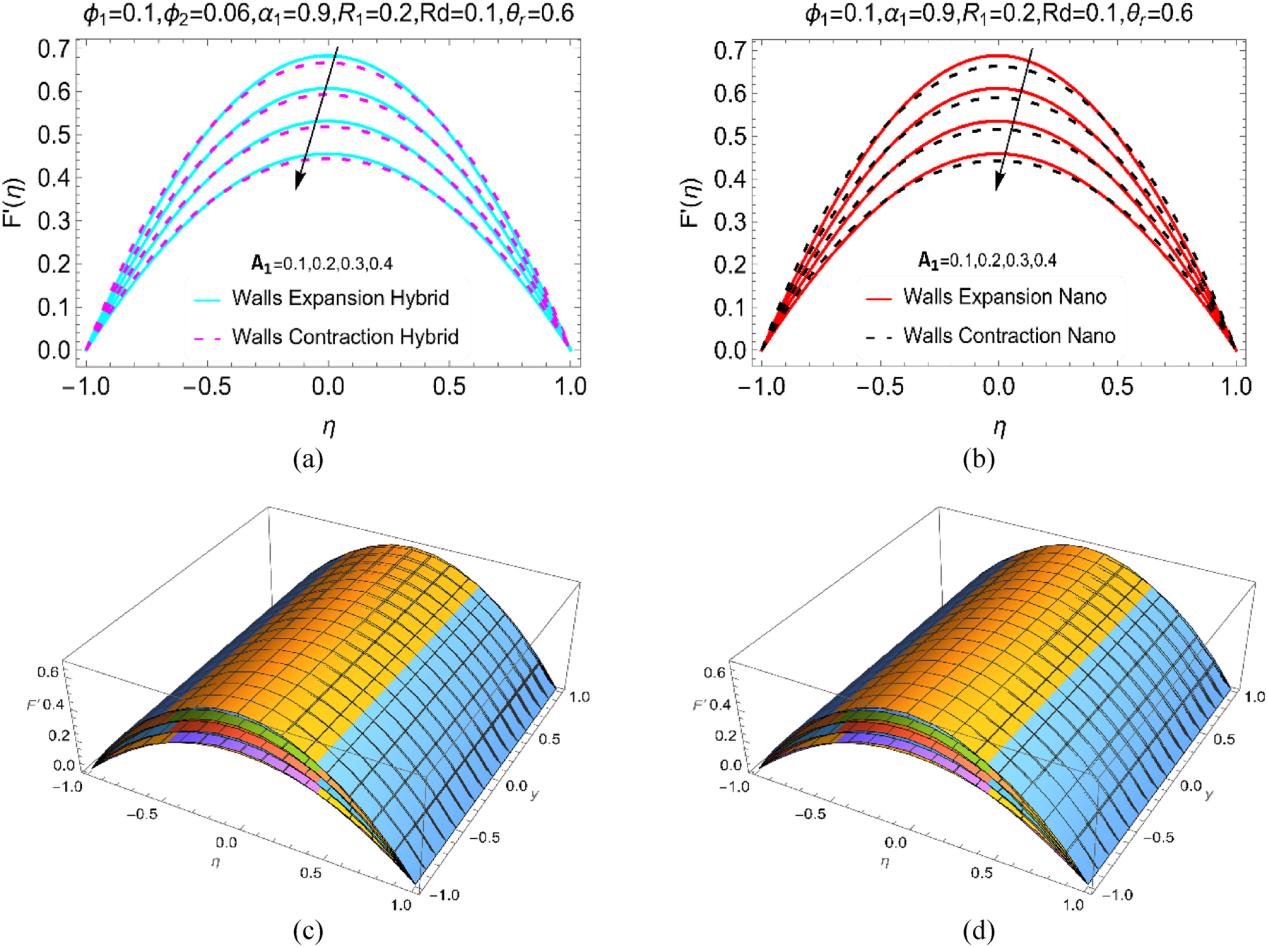
The velocity $\text{F}{^{\prime}}(\upeta )$ against ${\text{A}}_{1}$ for (**a**) Hybrid, (**b**) Nano and 3D view for (**c**) Hybrid and (**d**) Nano.

Figure 3a,b reveals the BHNF and BNF movement against the increasing values of the walls absorptivity (A_1_). The parallel results furnished for elongating and contracting walls. It can be observe that by enhancing the absorptivity of the walls, the fluid motion can be controlled. At the channel end, the movement of all the particles approaches to zero because of no slip effects there. For contraction of the walls, the particles loss their momentum as a consequence the motion declines. However, for expanding wall case the drop in the particles movement is slow. Physically, large working area between the channels produces due to expanding walls. Thus, the molecules of both BHNF and BNF can freely move there which lead to slow decrement in the motion. Further, for simple BNF, the fluid velocity drops quite rapidly compared to BHNF. To visualize the 3D behaviour of the fluid velocity $F{^{\prime}}(\eta )$, Fig. 3c,d furnished.

**Figure 4:**
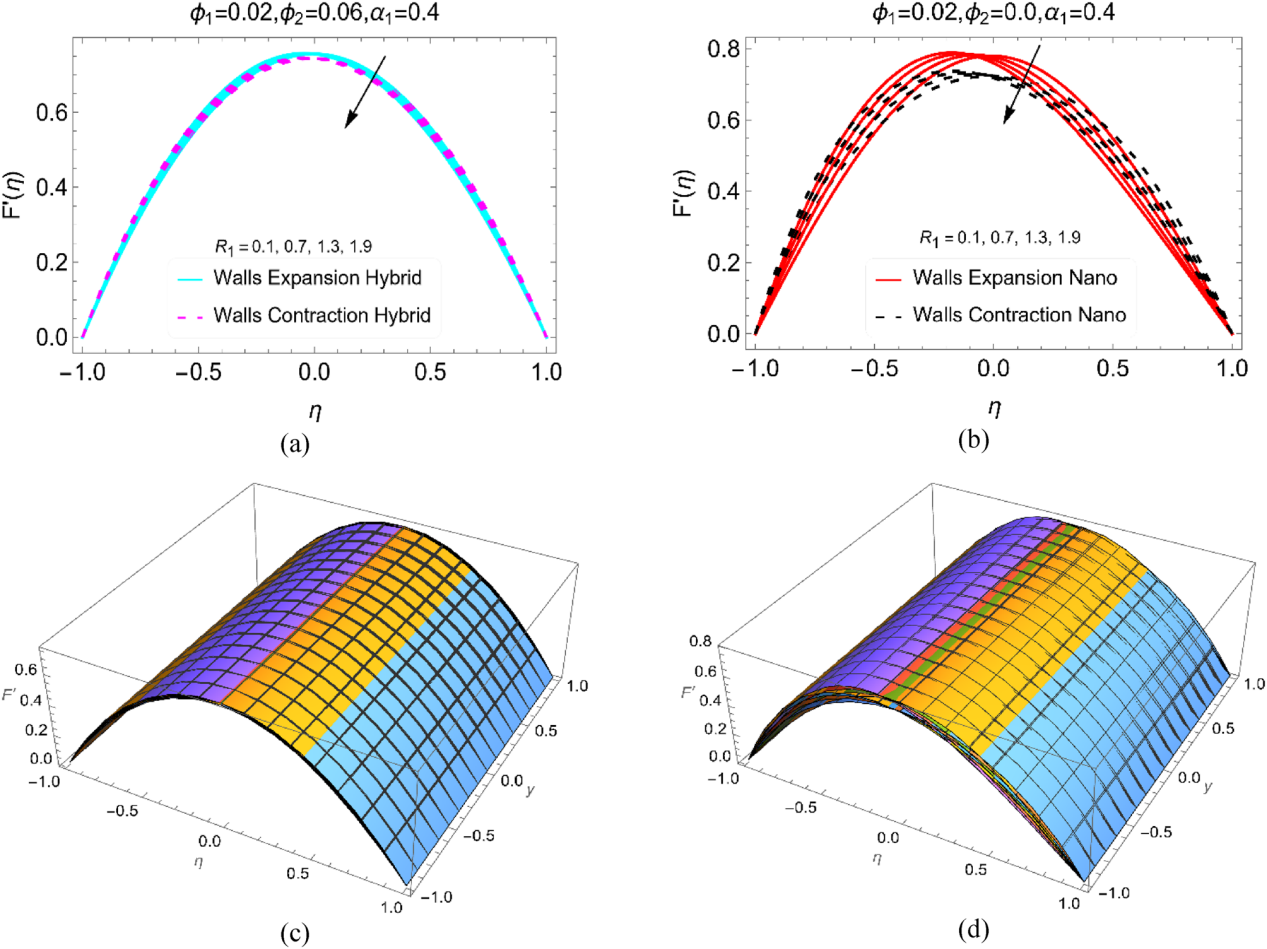
The velocity $\text{F}{^{\prime}}(\upeta )$ against ${\text{R}}_{1}$ for **(a)** Hybrid, **(b)** Nano and 3D view for (**c**) Hybrid and (**d**) Nano.

Reynolds number is one of the important dimensionless number which influence the fluid movement. To analyze the behaviour of $F{^{\prime}}$ against the enlarging values of ${R}_{1}$, Fig. 4a,b portrayed. In this study, the fluid movement decreases as the ${R}_{1}$ increases. It is obvious that the decrement occurs in the above half of the channel while it boosts in the lower half. However, for BHNF, these variations are very slow due to their dominant viscous forces. These forces are weaker in simple BNF, which allow the particles for rapid movement. Physically, it means that for BNF, the inertial forces are stronger then BHNF which are good for increase in the velocity. Further, Fig. 4c,d organized to inspect the 3D behavior of the velocity profiles furnished in Fig. 4a,b.

### Temperature of bionanofluids with changing parameters

**Figure 5:**
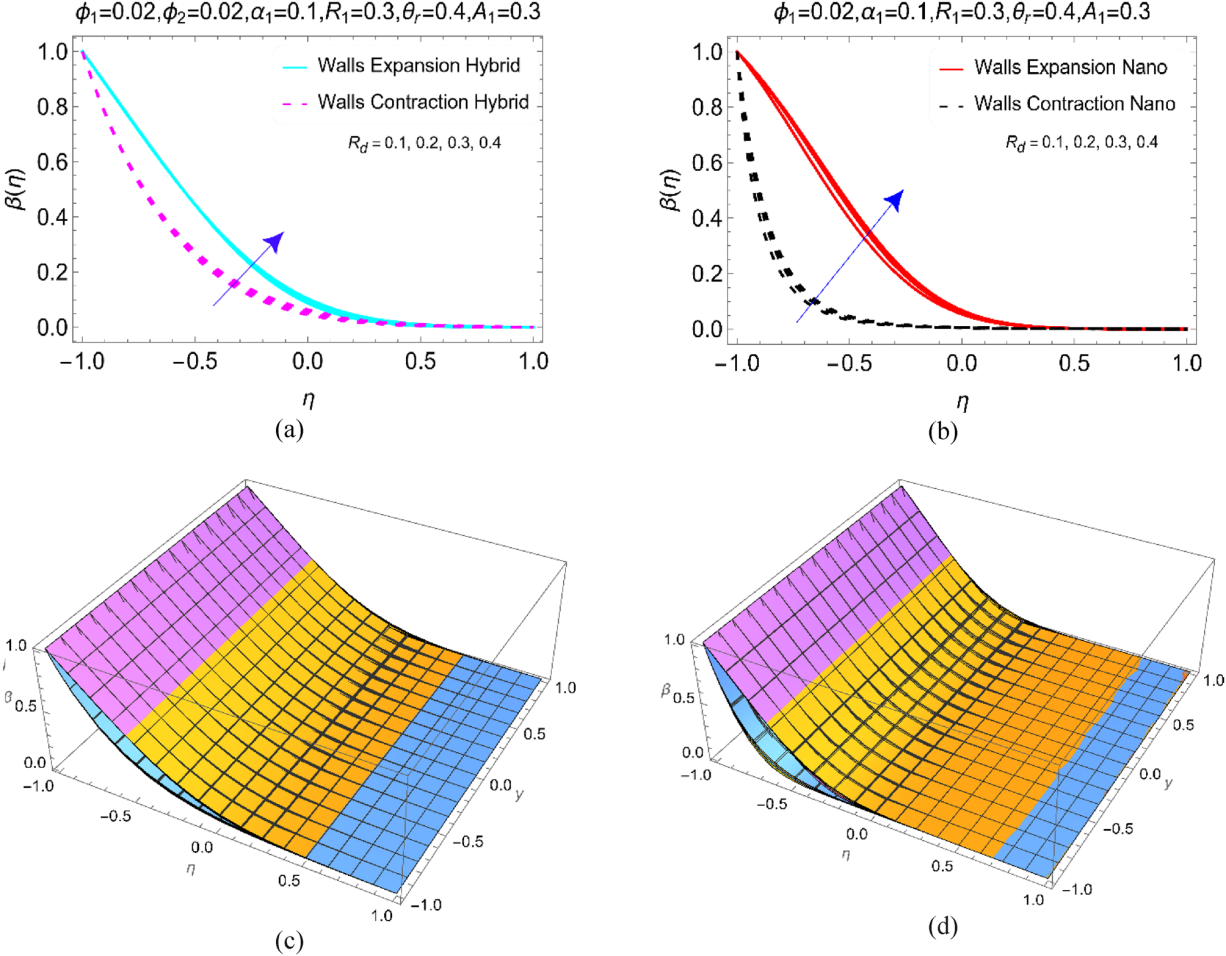
The temperature profile $\upbeta (\upeta )$ against ${\text{R}}_{\text{d}}$ for **(a)** Hybrid, **(b)** Nano and 3D view for (**c**) Hybrid and (**d**) Nano.

**Figure 6:**
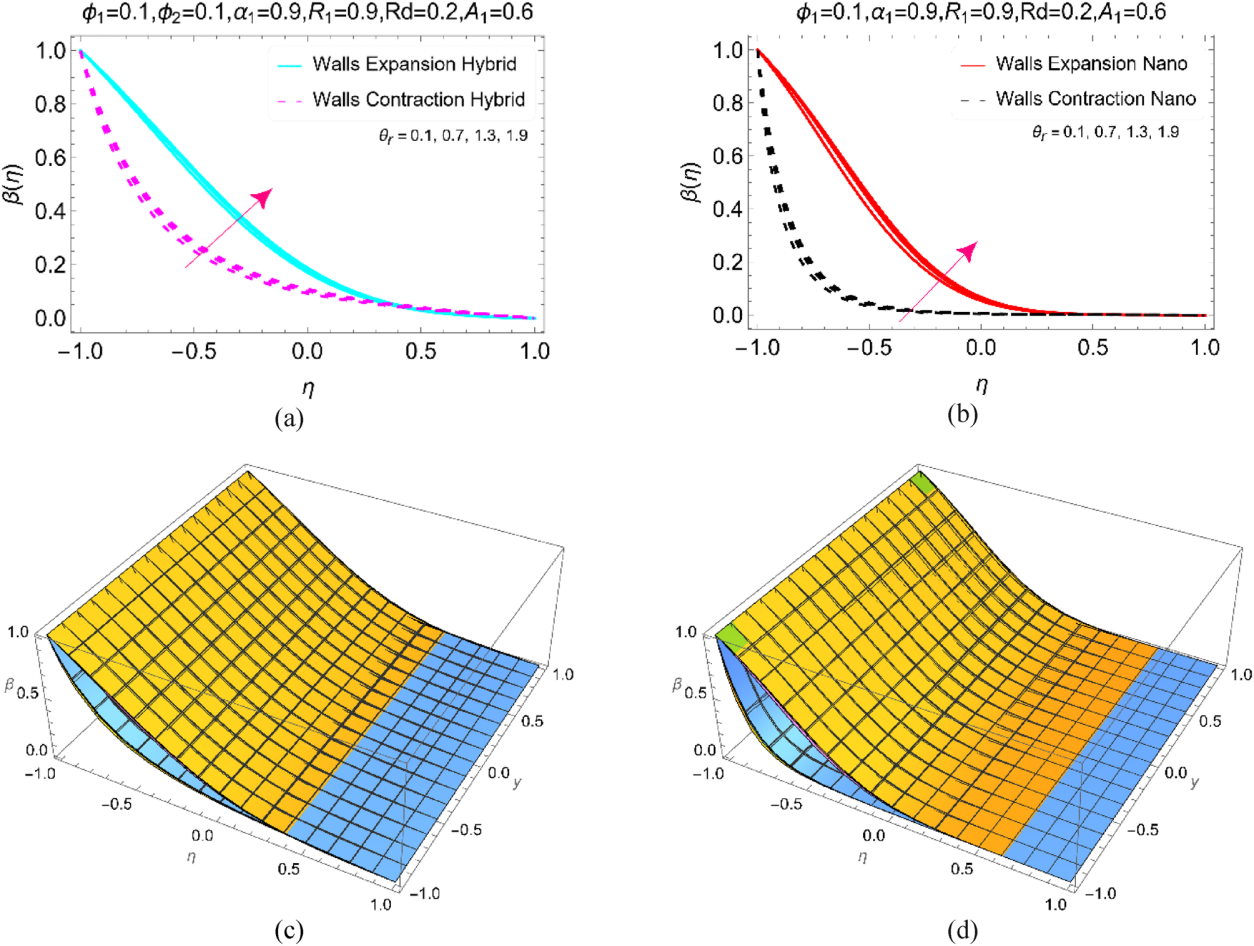
The temperature profile $\upbeta (\upeta )$ against ${\uptheta }_{\text{r}}$ for **(a)** Hybrid, **(b)** Nano and 3D view for (**c**) Hybrid and (**d**) Nano.

Thermal radiation ${R}_{d}$ and temperature ratio number ${\theta }_{r}$ effectively contribute in the temperature transmission of BHNF and BNF. For this purpose, Figs. 5 and 6 organized under multiple ranges. The impacts of quadratic radiation factor ${R}_{d}$ for elongating and contraction channel elaborated in Fig. 5a,b. Figure 5a deals with the heat performance of BHNF and Fig. 5b covers the effects for BNF. From these results it is examined that required heat transport in BHNF and BNF can be achieved by adding the radiations effects in the model. Expansion of the channel produces more heat in the fluid than that of contracting channel. Further, thermal boundary layer declines BNF for contraction case. The temperature of both BHNF and BNF vanishes towards to upper absorptivity wall. To examine the fascinating 3D view of Fig. 5a,b, the Fig. 5c,d is furnished for the same values of the radiative number as in Fig. 5a,b, respectively. It is of paramount interest that the value of Prandtl number ${P}_{r}$ is taken $21.0$ for blood.

Figure 6a,b reflects the temperature trends $\beta (\eta )$ under varying temperature ratio number ${\theta }_{r}$. This factor appeared due to the quadratic radiation and playing excellent role in the temperature of BHNF and BNF by considering the absorptivity and elongating/contraction of the walls. In both the working fluids, the temperature enhances when the values of ${\theta }_{r}$ increased from 0.1 to 1.9. Further, the 3D view can be visualized from Fig. 6c,d against the increasing ${\theta }_{r}$ as in Fig. 6a,b.

### Computation of skin friction and nusselt number

This subsection emphasis on the computational trends of the shear drag and local thermal gradient for (Graphene-CuO)/blood biohybrid nanofluid. The computation done for both top and bottom end of the channel. It is straightforward from Table 2 that the shear drag at the bottom end rises by adding more Graphene nanoparticles in blood. However, the shear drag can be optimized by contracting of the walls. Further, the absolute shear drag enhances at the top plate while the concentration varies and the other quantities kept constant as mentioned in the Table 2. The computation reveal that the viscosity number is a key to obtain maximum shear drag in comparison with all the other involved constraints.

**Table 2:** Computations for drag forces for biohybrid nanofluid at the bottom and top end of the channel.

Parametric ranges				Skin friction variations for biohybrid nanofluid (Graphene–CuO)/blood			
${\phi }_{1}$	${\phi }_{2}$	${R}_{1}$	${A}_{1}$	$F{^{\prime\prime}}(-1)$		$\left|{F}^{{{\prime\prime}}{^{\prime}}}\left(1\right)\right|$	
	$0.03$	$3.0$	$0.5$	Expanding case	Contracting case	Expanding case	Contracting case
$0.01$				$2.4193$	$2.5437$	$1.2515$	$1.3698$
$0.04$				$2.3134$	$2.4200$	$1.3155$	$1.4183$
$0.07$				$2.2409$	$2.3347$	$1.3643$	$1.4556$
$0.11$				$2.1739$	$2.2554$	$1.4137$	$1.4935$
$0.01$	$0.02$			$2.6633$	$2.8254$	$1.1313$	$1.2798$
	$0.04$			$2.2953$	$2.3988$	$1.3272$	$1.4273$
	$0.06$			$2.1709$	$2.2518$	$1.4160$	$1.4953$
	$0.08$			$2.1088$	$2.1777$	$1.4661$	$1.5340$
	$0.03$	$5.0$		$2.9355$	$3.0524$	$1.0770$	$1.1795$
		$7.0$		$3.4719$	$3.5828$	$0.9703$	$1.0578$
		$9.0$		$4.0156$	$4.1226$	$0.9034$	$0.9781$
		$11$		$4.5604$	$4.6650$	$0.8595$	$0.9239$
		$3.0$	$1.0$	$3.5616$	$3.7598$	$2.1357$	$2.3221$
			$1.5$	$2.8223$	$2.9716$	$1.5349$	$1.6761$
			$2.0$	$1.9917$	$2.0911$	$1.9793$	$1.0745$
			$2.5$	$1.0559$	$1.1053$	$0.4680$	$0.5161$

To investigate the local thermal gradient behavior of biohybrid nanofluid influenced by the model constraints, Table 3 organized. The intense analysis of the values indicate that increasing the particles concentration from 1.0 to 4.0% is good to execute the desired thermal gradient. However, at the top plate these values dominate. The temperature ratio number reduces the Nusselt number value in the particular study. Similarly, all the parametric trends set for the variations of Nusselt number and listed in Table 3.

**Table 3:** Computations Local thermal gradient for (Graphene-CuO)/blood bionanofluid at the bottom and top end of the channel.

Parametric ranges						Skin friction variations for biohybrid nanofluid (Graphene-CuO)/blood			
${\phi }_{1}$	${\phi }_{2}$	${R}_{1}$	${A}_{1}$	${R}_{d}$	${\theta }_{r}$	$\left|{\beta }^{^{\prime}}\left(-1\right)\right|$		$\left|\beta {^{\prime}}\left(1\right)\right|$	
	$0.03$	$0.3$	$0.5$	0.3	0.3	Expanding case	Contracting case	Expanding case	Contracting case
$0.01$						$0.8191$	$0.2117$	$0.2184$	$0.2580$
$0.02$						$0.1926$	$0.2114$	$0.2164$	$0.2450$
$0.03$						$0.1950$	$0.2106$	$0.2151$	$0.2374$
$0.04$						$0.1968$	$0.2103$	$0.2142$	$0.2325$
$0.01$	$0.02$					$0.1828$	$0.2126$	$0.2231$	$0.2904$
	$0.04$					$0.1929$	$0.2111$	$0.2231$	$0.2440$
	$0.06$					$0.1973$	$0.2103$	$0.2161$	$0.2312$
	$0.08$					$0.1997$	$0.2098$	$0.2138$	$0.2252$
	$0.03$	$0.1$				$0.1791$	$0.2004$	$0.2452$	$0.2886$
		$0.3$				$0.1891$	$0.2117$	$0.2184$	$0.2580$
		$0.5$				$0.1994$	$0.2234$	$0.1937$	$0.2296$
		$0.7$				$0.2100$	$0.2354$	$0.1710$	$0.2034$
		$0.3$	$0.1$			$0.1867$	$0.2091$	$0.2198$	$0.2596$
			$0.3$			$0.1914$	$0.2143$	$0.2171$	$0.2564$
			$0.5$			$0.1962$	$0.2196$	$0.2144$	$0.2533$
			$0.7$			$0.2010$	$0.2250$	$0.2118$	$0.2501$
			$0.5$	$0.2$		$0.2118$	$0.2376$	$0.1806$	$0.2055$
				$0.4$		$0.2040$	$0.2287$	$0.1950$	$0.2257$
				$0.6$		$0.1962$	$0.2196$	$0.2144$	$0.2533$
				$0.8$		$0.1882$	$0.2104$	$0.2419$	$0.2931$
				$0.3$	$0.1$	$0.1998$	$0.2234$	$0.2229$	$0.2620$
					$0.3$	$0.1962$	$0.2196$	$0.2144$	$0.2533$
					$0.5$	$0.1946$	$0.2190$	$0.2024$	$0.2402$
					$0.7$	$0.1977$	$0.2252$	$0.1858$	$0.2215$

### Model and code validation

The validity and authenticity of the code are presented in this subsection. As, the previously reported studies have been accomplished for Newtonian of up to nanofluids. However, the results of the current model can be authenticate. Therefore, the current model will compatible with the reported analysis when the particles concentration approaches to zero and by taken ${\alpha }_{1}=0.0$ and $M=0.0$. Thus, under these assumptions the study is compared and validated with the results of Bilal et al.^57^. The results enlisted in Table 4 are the evidence of the model and code validation.

**Table 4:** Comparison of the current computation with previous literature.

The current model results for $F{^{\prime}}{^{\prime}}(1)$ using VIM		
Parameter	Comparison	
${A}_{1}$	Bilal et al.^57^	Current computation
$0.5$	$4.713254$	$4.713253$

## Conclusions

This research examines the characteristics of BHNF and BNF in an expanding/contracting channel with permeable walls. The modeling has been done via BHNF properties and appropriate similarity equations and then investigated through VIM. Indepth analysis of the results shown that:

- The adverse pressure in the surrounding of the lower and upper plates opposes the BHNF and BNF velocity and favorable effects observed around $\eta =0.0$.
- The weaker viscous forces in BNF causes rapid movement than that of BHNF under increasing ${\alpha }_{1}$ for both expanding ($0.1 {\text{to}} 1.6$) and contracting ($- \, 0.1\text{ to}- \, 1.6$) cases.
- The high permeability of the walls i.e. ${A}_{1}=\text{0.1,0.2,0.3,0.4}$ reduces the motion of working fluid significantly.
- The radiative effects are good to enhance the temperature of BHNF and BNF keeping the Prandtl number as $21.0$.
- For more contracted walls, the velocity distribution reduced significantly.
- The heat transfer gradient increases rapidly when the average concentration of the particles taken as $1.0\%$ to $4.0\%$.

In future, the work could be extended for different hybrid nanofluids in the existence of controlling parameters like thermal and momentum slip and for small and large weight concentration of nanoparticles.

## Data Availability

The datasets used and/or analysed during the current study available from the corresponding author on reasonable request.

## List of symbols

$$\left[ {\tilde{u}, \tilde{v}} \right]$$: Velocity components (m/s)
$$\tilde{T}$$: Temperature (K)
$T_{l}$: Temperature at the lower plate (K)
$T_{u}$: Temperature at the upper plate (K)
$\rho_{biohnf}$: Density of BHNF (kg/m^3^)
$\mu_{biohnf}$: Dynamic viscosity of BHNF (kg/ms)
$k_{biohnf}$: Thermal conductivity of BHNF (W/mK)
$$\eta$$: Dimensionless variable
$$R_{1}$$: Reynolds number
$$A_{1}$$: Permeable number
$$R_{d}$$: Radiation number
$\theta_{r}$: Temperature coefficient number
$$F$$: Bionanofluids velocity
$$\beta$$: Bionanofluids temperature
